# Disability burden due to musculoskeletal conditions and low back pain in Australia: findings from GBD 2019

**DOI:** 10.1186/s12998-022-00434-4

**Published:** 2022-05-03

**Authors:** Katie de Luca, Andrew M. Briggs, Simon D. French, Manuela L. Ferreira, Marita Cross, Fiona Blyth, Lyn March

**Affiliations:** 1 Discipline of Chiropractic, School of Health, Medical and Applied Sciences, CQUniversity, Brisbane, Australia; 2 Department of Chiropractic, Faculty of Medicine, Health and Human Sciences, Macquarie University, Sydney, Australia; 3 Curtin School of Allied Health and Curtin enAble Institute, Faculty of Health Sciences, Curtin University, Perth, Australia; 4 Global Alliance for Musculoskeletal Health, Sydney, Australia; 5 Institute of Bone and Joint Research, Kolling Institute, Faculty of Medicine and Health, University of Sydney, Sydney, Australia; 6 School of Public Health, Faculty of Medicine and Health, University of Sydney, Sydney, Australia; 7 Florance and Cope Professorial Department of Rheumatology, Royal North Shore Hospital, Sydney, Australia

**Keywords:** Musculoskeletal, Low back pain, Disease burden, Burden, Health policy

## Abstract

**Background:**

To report the national prevalence, years lived with disability (YLDs) and attributable risk factors for all musculoskeletal conditions and separately for low back pain (LBP), as well as compare the disability burden related to musculoskeletal with other health conditions in Australia in 2019.

**Methods:**

Global Burden of Disease (GBD) 2019 study meta-data on all musculoskeletal conditions and LBP specifically were accessed and aggregated. Counts and age-standardised rates, for both sexes and across all ages, for prevalence, YLDs and attributable risk factors are reported.

**Results:**

In 2019, musculoskeletal conditions were estimated to be the leading cause of YLDs in Australia (20.1%). There were 7,219,894.5 (95% UI: 6,847,113–7,616,567) prevalent cases of musculoskeletal conditions and 685,363 (95% UI: 487,722–921,471) YLDs due to musculoskeletal conditions. There were 2,676,192 (95% UI: 2,339,327–3,061,066) prevalent cases of LBP and 298,624 (95% UI: 209,364–402,395) YLDs due to LBP. LBP was attributed to 44% of YLDs due to musculoskeletal conditions. In 2019, 22.3% and 39.8% of YLDs due to musculoskeletal conditions and LBP, respectively, were attributed to modifiable GBD risk factors.

**Conclusions:**

The ongoing high burden due to musculoskeletal conditions impacts Australians across the life course, and in particular females and older Australians. Strategies for integrative and organisational interventions in the Australian healthcare system should support high-value care and address key modifiable risk factors for disability such as smoking, occupational ergonomic factors and obesity.

**Supplementary Information:**

The online version contains supplementary material available at 10.1186/s12998-022-00434-4.

## Introduction

Musculoskeletal conditions include more than 150 discrete conditions, and these conditions are highly prevalent, affecting 1.7 billion people globally [1]. In Australia, musculoskeletal conditions have been reported to account for 25% of non-fatal disease burden, with low back pain (LBP) the leading individual cause [2]. LBP is highly prevalent, with 70–90% of people suffering from LBP at some point in their lives [3]. As a result, LBP costs the Australian healthcare system AUD$4.8 billion annually [4], is one of the most common reasons for visiting a general practitioner [5], and is the leading cause of lost productive years [6]. With approximately 50% of people having disability beyond 3 months, LBP leads to poorer quality of life, psychological distress, and disability [7]. Therefore, with compelling evidence that musculoskeletal conditions impose a substantial personal and societal burden, attention is needed to further understand the burden of musculoskeletal conditions, and in particular for LBP, at a national level in Australia.

The Global Burden of Disease (GBD) study systematically quantifies health loss for more than 350 diseases by age, sex, year, and geographic location, and allows for the comparison of burden across disparate diseases over time [8]. The GBD study allows for the assessment of national population health as a means to inform national and local policy decisions and resource allocation. Outside of the global GBD capstone publications for musculoskeletal conditions [9–13], estimates for musculoskeletal conditions have been summarised and interpreted specifically for individual countries [14–16]. As knowledge translation and visualisations of GBD data engages users and is seen as valuable by policymakers [17, 18], national-level musculoskeletal disease burden estimates and temporal trends may enable local health system improvement and policy evolution. Contextualising GBD 2019 musculoskeletal estimates will provide clinicians, researchers and Australian health policy makers with comprehensive and comparable health estimates and will allow for common disability burden metrics to be compared with other countries.

The aim of this systematic analysis is to report the national prevalence, years lived with disability (YLDs) and attributable risk factors for “all” musculoskeletal conditions combined (LBP; neck pain; hip, knee, hand and other osteoarthritis; rheumatoid arthritis; gout; and a group of other musculoskeletal conditions) and separately for LBP, as well as compare the disability burden related to musculoskeletal with other health conditions in Australia in 2019.

## Methods

### Overview

The detailed approach to estimating causes of death and disease incidence and prevalence for GBD 2019 is reported elsewhere [8]. The GBD study uses a systematic method to analyse fatal and non-fatal trends across countries for diseases and injuries. Data are derived from the GBD repository of population health data. For most diseases and injuries, processed data are modelled using standardised tools to generate estimates of each quantity of interest by age, sex, location, and year. DisMod-MR is a Bayesian meta-regression tool that allows evaluation of all available data for a disease, ensuring consistency between epidemiological parameters. A flowchart of variables put into the DisMod-MR model can be found in Vos et al., Appendix 1, p.1198 [8]. For nonfatal health outcomes, there were 14 input sources for all musculoskeletal conditions and 12 input sources for LBP in Australia. Observed Australian data input sources for estimating the burden of musculoskeletal conditions and LBP can be found at: https://ghdx.healthdata.org/gbd-2019/data-input-sources?components=5&causes=626&locations=71.

Disability adjusted life years (DALYs) is the standard metric used to quantify burden and is calculated by combining years of life lost (YLLs) due to premature mortality and YLDs, with one DALY considered as one lost year of "healthy" life. As disability is the primary impact of musculoskeletal conditions, and there is no direct mortality related to LBP, in this paper YLDs are reported to quantify burden, specifically disability burden. YLDs were calculated by multiplying the prevalence of a sequela by the disability weight for the corresponding health state. GBD 2010 and GBD 2019 lay descriptions and disability weights for LBP, with proportions within severity level for LBP, can be found in Additional files 1 and 2 [8]. The average disability weight was multiplied by the age/sex/region-specific prevalence to derive YLDs and uncertainty was incorporated by sampling 1000 draws at each computational step. The GBD Study adheres to the Guidelines for Accurate and Transparent Health Estimates Reporting (GATHER) statement [19].

Established case definitions for musculoskeletal conditions have been previously described [20–22]. GBD 2019 reported health estimates for “all” musculoskeletal conditions, including: LBP; neck pain; hip, knee, hand and other osteoarthritis; rheumatoid arthritis; gout; and a group of other musculoskeletal conditions. The category of “other” refers to a wide range of autoimmune, inflammatory, joint, ligament, tendon, and muscle disorders that vary across epidemiologic studies. Prevalence and burden estimates for all musculoskeletal conditions [LBP; neck pain; hip, knee, hand and other osteoarthritis; rheumatoid arthritis; gout and other musculoskeletal conditions combined (level 2 detail)], and for LBP (level 3 detail) are reported in this paper. Estimates are presented in terms of prevalence and disability estimates (expressed as YLDs), in terms of numbers (count) and age standardised rates (ASRs) (per 100,000 population), with corresponding 95% uncertainty intervals (UIs) [23, 24].

### Data access and aggregation

The meta-data on all musculoskeletal conditions and LBP burden in Australia are publicly available from GBD 2019, and shared, where legally permissible, through Institute for Health Metrics and Evaluation’s (IHME) Global Health Data Exchange (gHDX) (http://ghdx.healthdata.org/gbd-2019). From November 2020 to March 2021, meta-data and visualisations were accessed from gHDX, aggregated, interpreted and presented in this report. The GBD Compare tool was used to rank all health conditions in Australia and allowed for comparisons of change from 1990 to 2019.

### Attributable burden

GBD 2019 meta-data provided proportions of all musculoskeletal conditions and LBP burden-attributable risk factors in the following categories: behavioural (smoking); metabolic [high body mass index (BMI)] and impaired kidney function); environmental factors (occupational ergonomic); and the overlap between behavioural, metabolic, and environmental risk factors. For LBP, risk factors for which there was judged to be enough data to establish a relationship were high BMI, smoking, and occupational ergonomic factors. Additional details can be found in the GBD 2019 risk factor publication [25].

## Results

### Prevalence, burden and age and sex patterns of musculoskeletal conditions in Australia in 2019

**Fig. 1:**
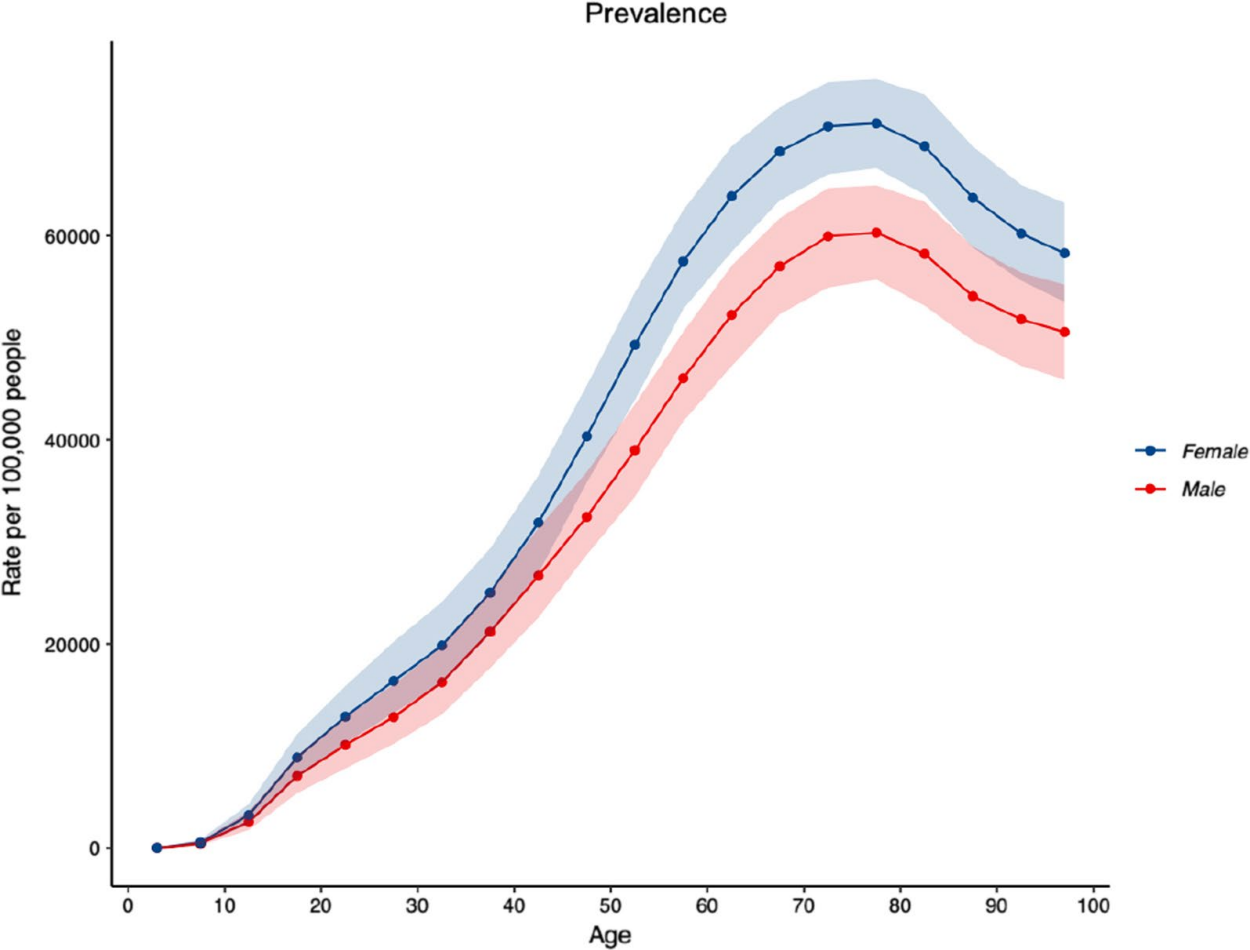
Age-standardised rates of prevalence for “all” musculoskeletal conditions (low back pain, neck pain, osteoarthritis (hip, knee, hand and other), rheumatoid arthritis, gout, and other musculoskeletal conditions), by age, for both sexes, per 100,000 population in Australia in 2019

2019 prevalence and YLD counts and ASRs per 100,000, and the corresponding percentage change in ASRs per 100,000 for prevalence and YLDs between 1990 and 2019 for all musculoskeletal conditions are shown in Table 1. The number of prevalent cases of musculoskeletal conditions was estimated at 7,219,894.5 (95% UI: 6,847,113.0–7,616,567.8). The ASR for prevalence per 100,000 population was 21,960.5 (95% UI: 20,743.3–23,247.7), an increase of 5.0% (95% UI: 2.1–8.0) since 1990. People aged 75–79 years of age had the highest ASR for prevalence per 100,000 population (65,980.8; 95% UI: 61,690.2–70,278.5) (Fig. 1). The ASR for prevalence per 100,000 population for all musculoskeletal conditions was higher in females (24,096.7; 95% UI: 22,726.9 to 25,534.3), compared to males (19,718.7; 95% UI: 18,570.9–20,906.8).

**Table 1:** Prevalence and years lived with disability counts, age-standardised rates per 100,000 population and the corresponding percentage change in age-standardised rates per 100,000 population between 1990 and 2019 for “all” musculoskeletal conditions in Australia

Prevalent cases			YLD		
2019 counts	2019 ASR	% change in ASR (‘90–‘19)	2019 counts	2019 ASR	% change in ASR (‘90–‘19)
7,219,893.5 (6,847,113.0–7,616,567.8)	21,960.5 (20,743.3–23,247.7)	5.1 (2.1–8.0)	685,363.3 (487,722.7–921,471.5)	2132.6 (1522.7–2854.7)	1.8 (− 2.5 to 5.7)

95% uncertainty intervals are in parentheses; ASRs as per 100,000 population

“All” musculoskeletal conditions combined, includes low back pain, neck pain, osteoarthritis (hip, knee, hand and other), rheumatoid arthritis, gout, and other musculoskeletal conditions

*ASR* age standardised rate, *YLD* years lived with disability

**Fig. 2:**
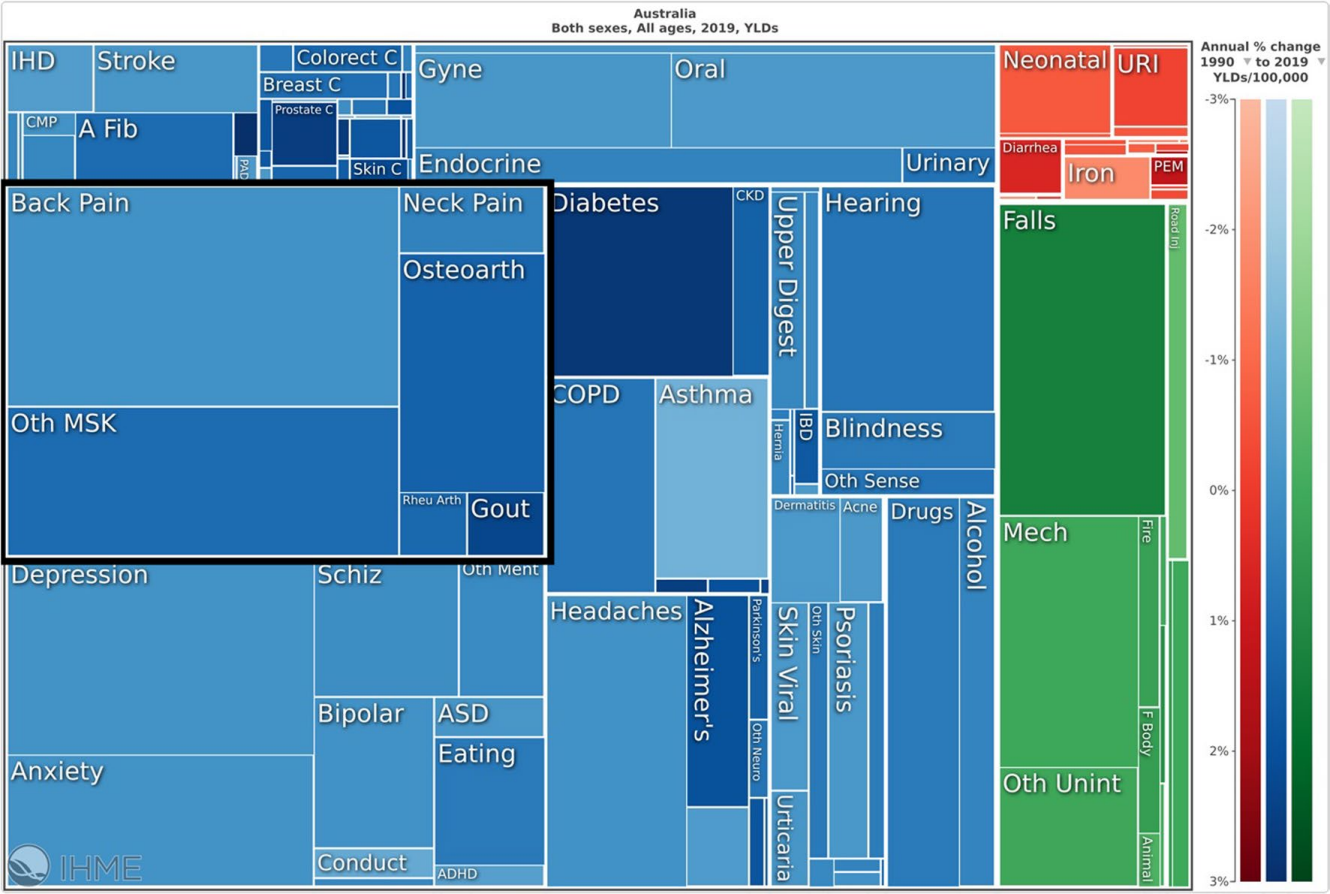
Proportion of years lived with disability, due to “all” musculoskeletal conditions (low back pain, neck pain, osteoarthritis (hip, knee, hand and other), rheumatoid arthritis, gout, and other musculoskeletal conditions) for both sexes, in Australia in 2019

**Fig. 3:**
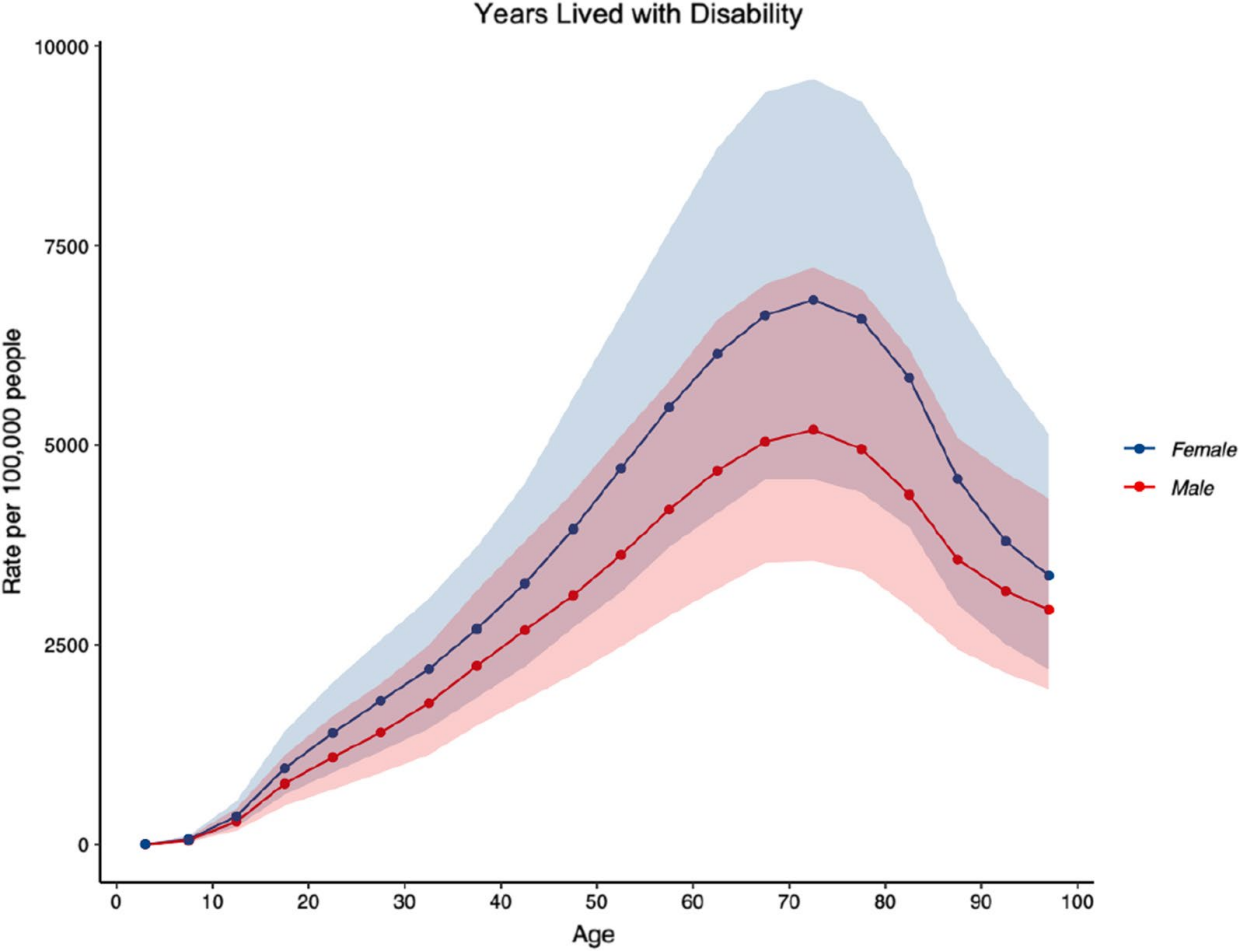
Age-standardised rates of years lived with disability for “all” musculoskeletal conditions (low back pain, neck pain, osteoarthritis (hip, knee, hand and other), rheumatoid arthritis, gout, and other musculoskeletal conditions), by age, for both sexes, per 100,000 population in Australia in 2019

Musculoskeletal conditions accounted for 20.1% (95% UI: 17.4–23.3%) of the total YLDs (Fig. 2) and 11.1% (95% UI: 9.1–13.4%) of the total DALYs in Australia, and were ranked first, ahead of mental disorders (17.8; 95% UI: 14.8–20.5%) and unintentional injuries (11.7; 95% UI: 9.7–14.2%). Table 2 shows the top 10 rankings of all-age YLDs for non-communicable diseases in 1990 and 2019 for both sexes, showing percent change of all-age counts and age-standardized rates. In 2019, the number of YLDs due to all musculoskeletal conditions was 685,363.3 (95% UI: 487,722.7–921,471.5). ASRs of YLDs per 100,000 population due to all musculoskeletal conditions were 2,132.6 (95% UI: 1,522.7–2,854.7). Between 1990 and 2019, for both sexes and across all ages, there were increases in counts for YLDs of 75.3% (95% UI: 66–84, and ASRs per 100,000 population for YLDs of 1.8% (95% UI: − 2.45 to 5.8). People aged 70–74 years having the highest YLD rate (6026.8; 95% UI: 4136.7–8402.0) (Fig. 3). The ASR for YLD per 100,000 population was higher in women (2385.8; 95% UI: 1702.5–3194.6) compared to men (1866.6; 95% UI: 1333.0–2479.1).

**Table 2:**
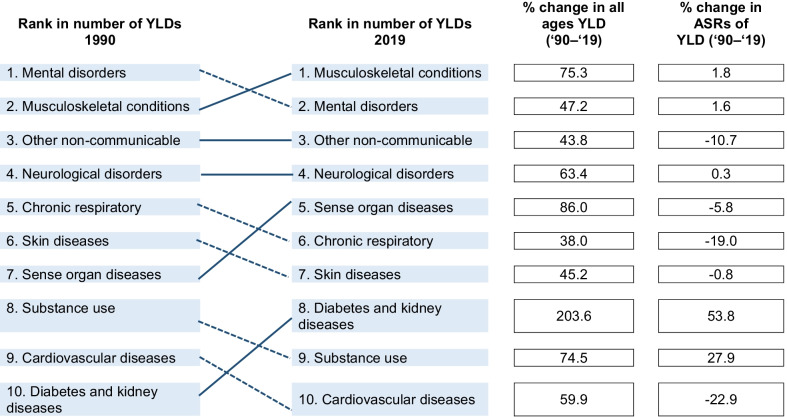
Rank of years lived with disability (YLD) for non-communicable diseases in Australia, in 1990 and 2019 for both sexes and across all ages, showing percent change of counts and age-standardized rates (ASRs) per 100 000 population

*The line connecting a box for a disease or injury type in 1 year to the corresponding box in a later year is solid if the rank rose or did not change; the line is dashed if the rank declined

### Prevalence, burden and age and sex patterns of low back pain in Australia in 2019

**Fig. 4:**
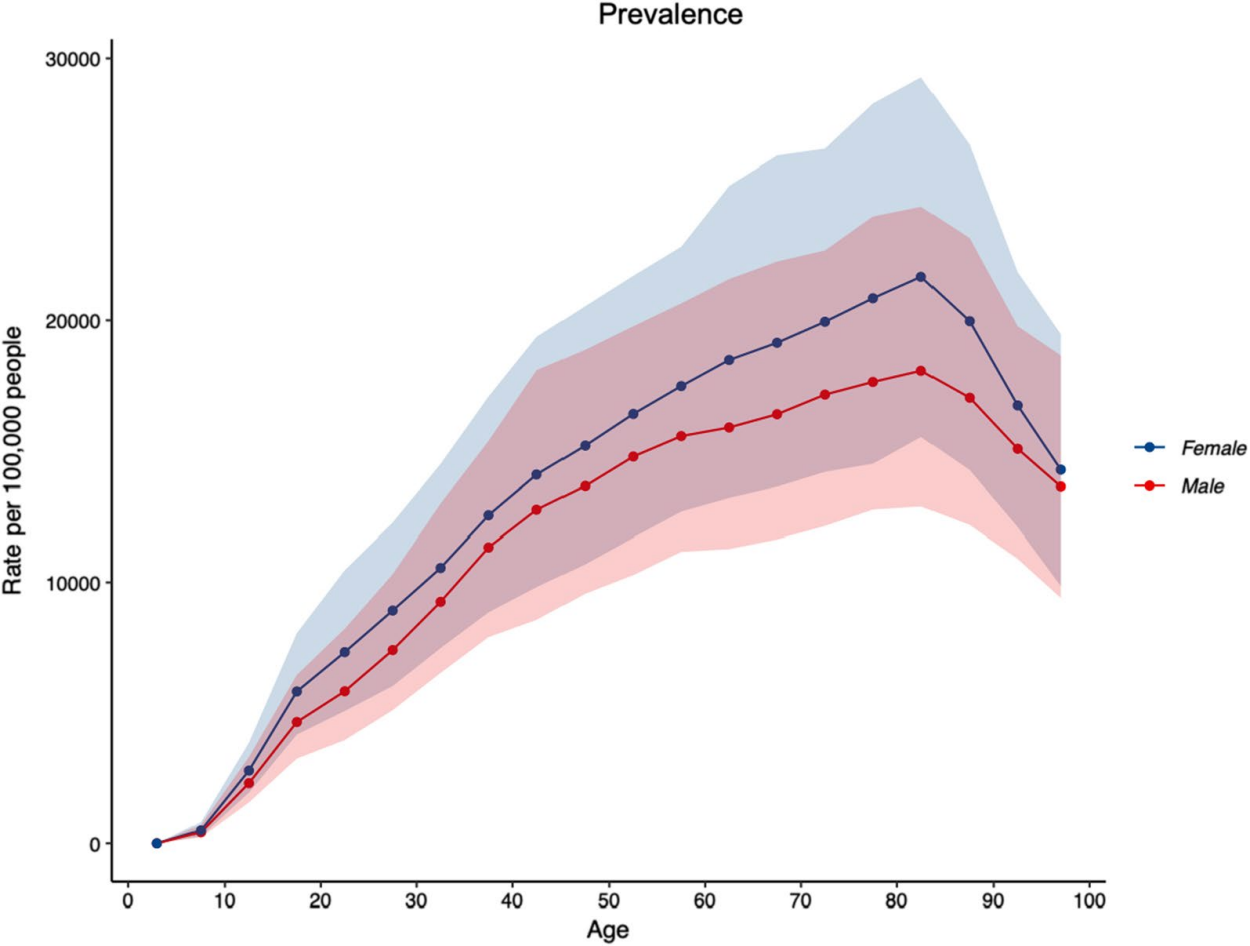
Age-standardised rates of prevalence for low back pain, by age, for both sexes, per 100,000 population in Australia in 2019

**Fig. 5:**
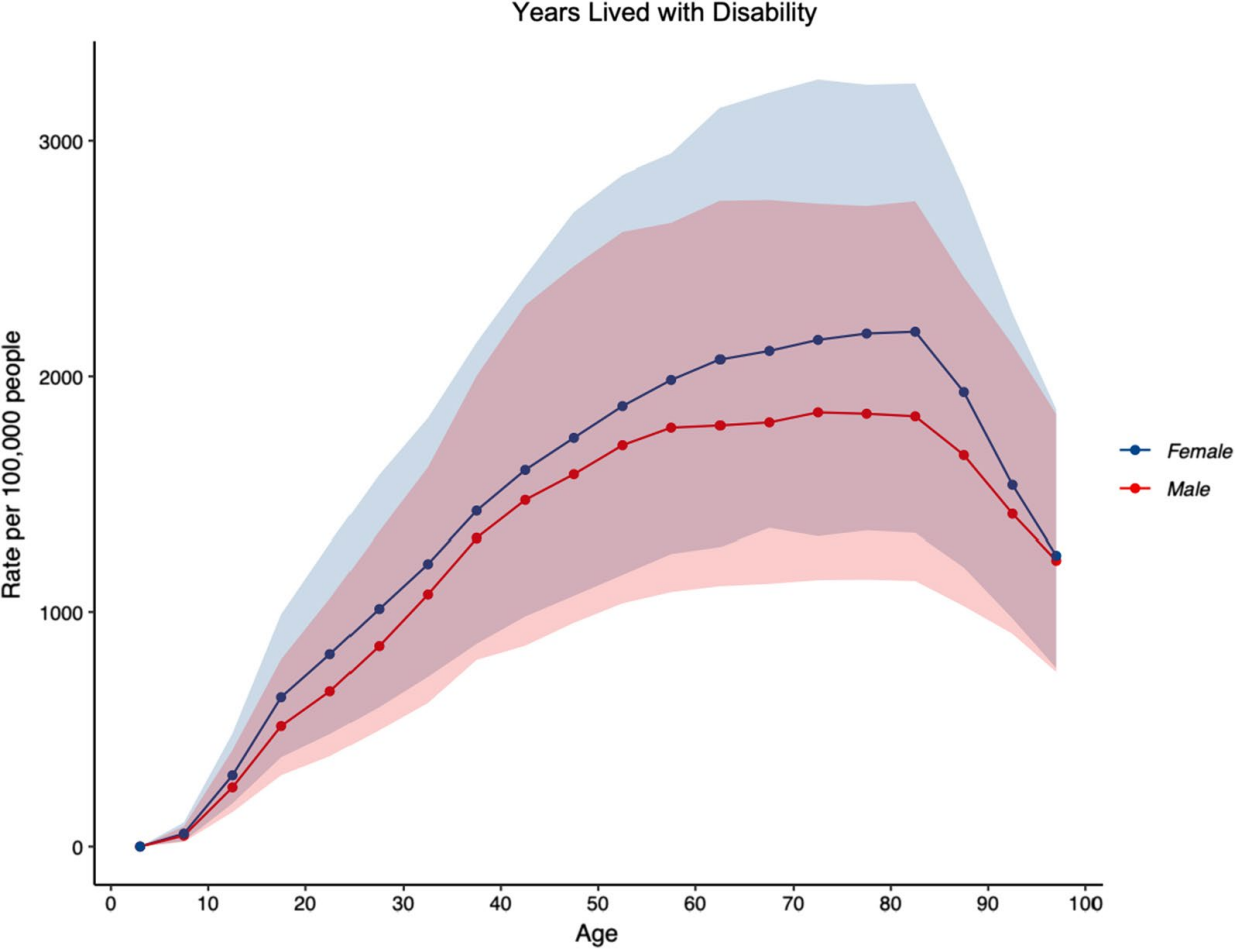
Age-standardised rates of years lived with disability for low back pain, by age, for both sexes, per 100,000 population in Australia in 2019

2019 prevalence and YLD counts and ASRs per 100,000, and the corresponding percentage change in ASRs per 100,000 for prevalence and YLDs between 1990 and 2019 for LBP are shown in Table 3. LBP accounted for a substantial proportion of YLDs attributed to all musculoskeletal conditions (44%). People aged 80–84 years of age had the highest ASR of prevalence per 100,000 population (20,046.8; 95% UI: 14,310.7–26,763.1) (Fig. 4) and the highest YLD rate (2028.5; 95% UI: 1275.3–3006.6) for LBP (Fig. 5). ASR for prevalence per 100,000 population for LBP was higher in females (9396.1; 95% UI: 8175.0–10,761.6), compared to males (8152.3; 95% UI: 7048.7–9395.6), and the ASR for YLD per 100,000 population was also higher in females (1049.8; 95% UI: 728.6–1413.9), compared to males (921.0; 95% UI: 639.5–1250.2).

**Table 3:** Prevalence and years lived with disability counts, age-standardised rates per 100,000 population and the corresponding percentage change in age-standardised rates per 100,000 population between 1990 and 2019 for low back pain in Australia

Prevalent cases			YLD			
2019 counts	2019 ASR	% change in ASR (‘90–‘19)	2019 counts		2019 ASR	% change in ASR (‘90–‘19)
2,676,192 (2,339,327–3,061,066)	8785.5 (7641.1 to 10,088.8)	− 10.2 (− 15.9 to − 4.7)	298,624 (209,364–402,395)		986.6 (685.3–1334.1)	− 11.4 (− 17.1 to − 6.0)

95% uncertainty intervals are in parentheses; ASRs as per 100,000 population

*ASR* age standardised rate, *YLD* years lived with disability

### Attributable risk factors

Across all ages and both sexes, attributable modifiable GBD risk factors accounted for 22.3% (95% UI: 18.4–26.2) of YLDs due to all musculoskeletal conditions (9% high BMI and 9% smoking, 7.3% occupational ergonomic factors and 0.5% kidney dysfunction). Across all ages and both sexes, attributable modifiable GBD risk factors accounted for 39.8% (95% UI: 34.9–45.0) of YLDs due to LBP (20.1% smoking, 16.6% occupational ergonomic factors and high BMI 10.6%).

## Discussion

In Australia in 2019, musculoskeletal conditions were estimated to be the leading cause of disability burden, ahead of both mental health and unintentional injuries, and were responsible for one fifth of the total YLDs. ASRs for prevalence and disability burden were high across the life course, peaking in older adults and being higher in females. LBP was the most prevalent musculoskeletal condition and was the leading driver of disability burden in Australia. These data show the impact of LBP on the Australian community and highlight the importance of increased societal and governmental awareness of LBP-related disability [26].

Estimates of prevalence and burden of disease are important tools for monitoring population health, with both national and global studies useful in interpreting available data for different contexts. There are small variations between estimates for overall burden of musculoskeletal conditions modelled in GBD 2019 (11%) and that reported by the Australian Institute of Health and Welfare (AIHW) in 2018 (13%) [27]. Similarly, GBD estimates for non-fatal disease burden were slightly lower (20%) than Australian Burden of Disease Study (ABDS) 2018 data (24%). Some indirectness may be found in the Australian GBD musculoskeletal and LBP estimates due to the data input sources, i.e. no GBD 2019 data input sources can be retrieved for neck pain and rheumatoid arthritis in Australia between 1990 and 2019, and no direct input prevalence study appears for LBP since 2012. Therefore, the use of direct national data for a whole population may be more accurate. Also, in a comparison of rank and YLDs estimates between ABDS and GBD for 2015, differences have been found for rheumatoid arthritis and osteoarthritis [28]. Higher ABDS ranks and YLDs estimates may be due to representative Australian data sources and severity distributions with higher proportions of moderate and severe rheumatoid arthritis and osteoarthritis [28]. Key differentials that exist for GBD modelled data, is that the methodological approach used enables the reporting of trends over time and comparability across countries.

Musculoskeletal conditions are a significant health problem requiring rehabilitation across the life course [1]. GBD 2019 data reveal that LBP prevalence was high in all age groups from 15 years onwards, with ABDS 2018 data revealing back pain and problems were ranked second in terms of disease burden (DALYs) from 25 to 74 years [27]. Representative cohort data show that LBP experienced by Australian adolescents is associated with care seeking, medication use, school absenteeism, and reduced health-related quality of life [29]. As LBP is one of only four causes that drive increases in the number of DALYs from teenage years into older age [8], interventions focused on the prevention of musculoskeletal conditions, particularly LBP, through diet, exercise, lifestyle and workplace factors, and new models of care to improve access to appropriate health care for younger Australians, need urgent evaluation. Females and older adults have a higher prevalence of musculoskeletal conditions (in 24% of women compared to 19% of men), with two thirds of people aged 75–79 years having a musculoskeletal condition. Furthermore, in Australia, women aged 35–74 years’ experience 21% of their total disease burden from musculoskeletal conditions, compared to 14% for men of the same age group [27]. Older adults often do not receive optimal management of their LBP, even though their LBP is more disabling than that seen in younger adults [30]. Without appropriate management, musculoskeletal conditions can culminate in increased cognitive decline, sarcopenia, frailty, loss of independence, as well as long-term and expensive health care needs [31]. The UN “Decade of Healthy Ageing” has been designated for 2021–2030. Alongside a global, health systems framework for improving musculoskeletal health [26], a strong Australian platform is needed to strengthen local and relevant health initiatives that not only decrease musculoskeletal burden but also improve function (including work participation and functional independence), quality of life and reduce health inequity across the life course.

A number of GBD modifiable risk factors (smoking, ergonomic occupational factors and obesity) account for a sizable proportion of LBP disability burden. People with back problems have been shown to be more likely to be current or former smokers [7] and smoking has been associated with both higher lifetime risk of musculoskeletal pain [32], and higher pain intensity [33]. Cross-sectoral health promotion programs which target smoking and LBP could be integrated with other public health messaging for non-communicable diseases. Objectively measured sitting [34], along with lifting intensity and frequency at work [35], have also been associated with LBP. With 32% of workers not back at work after 1 month, it has been suggested that interventions targeting individual-level risk factors should be implemented soon after injury to prevent long-term work absence [36]. The transition rate from acute to chronic LBP is significantly higher in obese adults, smokers, those with severe and very severe baseline disability and in adults diagnosed depression/anxiety, with biomechanical, psychological and psychosocial factors identified as prognostic factors for the development of chronic back pain [37]. In particular, depressive symptoms are a significant risk factor for the report of future back pain in older adults [38]. Primary and secondary prevention strategies that integrate modifiable risk factors, such as smoking, intense and strenuous physical work environments and obesity are needed in the broader context of high-value care approaches to effectively reduce the burden of disease associated with musculoskeletal conditions and LBP.

Age standardised rates of YLDs due to musculoskeletal conditions increased by 3.2% from 1990 to 2019, with the ABDS 2018 reporting a 7.3% increase in burden from back pain and problems from 2003 to 2018 [27]. With disease burden continuing to increase over time, there is a timely need for a whole-of-system effort in optimising prevention and management. While state-level Models of Care for musculoskeletal conditions have been developed and are at varying stages of implementation and evaluation [39], the profound disease burden due to LBP remains. A number of health system factors contribute to this problem, in particular service and funding models that support low-value approaches and inadequately support high-value care. Recommendations from high-quality clinical practice guidelines suggests that high-value care includes patient centered care, assessing psychosocial factors, providing education/information, addressing physical activity/exercise and trying to keep patients at work [40]. Furthermore, implementation of the National Strategic Action Plan for Pain Management [41] will be an important catalyst to whole-of-system improvements to prevention and management of musculoskeletal pain conditions.

## Limitations

The estimates in all GBD analyses are modelled, rather than original data due to the lack of complete coverage of country level data globally. As GBD estimates both the severity and severity distributions of LBP from healthcare-seeking individuals in the US, they may not be generalisable to the general population with LBP. Further, severity distributions are likely to differ between countries due to issues such as differences in access to health care, availability and affordability of medications, and social and cultural environments, and results may not be reliable in the Australian setting. Internal and external validity of the GBD 2019 LBP disability weights and severity distributions are unknown. Musculoskeletal conditions, and particularly LBP [42], are less likely than other chronic diseases to have country level data, partly due to their low level of associated mortality. While, modelled estimates aim to adjust for the variable coverage, the heterogeneity of case definitions and the lack of standardisation of data collection, imprecision often exists as seen in the uncertainty in YLDs due to all MSK causes. To facilitate future capture of national level data, the Global Alliance for Musculoskeletal Health (G-MUSC) and the GBD 2010 Musculoskeletal Expert Group have developed a standardised survey questionnaire for measuring the population prevalence of LBP and other musculoskeletal conditions [43]. It is suggested that, this survey questionnaire be utilised in future population-based studies, with an emphasis placed on rural, regional, indigenous and vulnerable communities who may be the most in need of public health care interventions.

## Conclusion

The ongoing high burden due to musculoskeletal conditions impacts Australians across the life course, and in particular females and older Australians. Strategies for integrative and organisational interventions in the Australian healthcare system should support high-value care and address key modifiable risk factors for disability such as smoking, occupational ergonomic factors and obesity.

## Supplementary Information

**Additional file 1**. GBD lay descriptions and disability weights for low back pain in 2010 and 2019.

**Additional file 2**. Proportions within severity level, split by with and without leg pain, for low back pain in GBD 2010 and GBD 2019.

## Data Availability

Data aggregated and contextualised during the current study are publicly available from GBD 2019, and shared, where legally permissible, through Institute for Health Metrics and Evaluation’s (IHME) Global Health Data Exchange (gHDX) (http://ghdx.healthdata.org/gbd-2019).

## Abbreviations

ABDS: Australian Burden of Disease Study
AIHW: Australian Institute of Health and Welfare
ASRs: Age standardised rates
BMI: Body mass index
DALYs: Disability adjusted life years
GATHER: Guidelines for Accurate and Transparent Health Estimates Reporting
GBD: Global Burden of Disease
gHDX: Global Health Data Exchange
IHME: Institute for Health Metrics and Evaluation’s
LBP: Low back pain
NCDs: Non-communicable diseases
UI: Uncertainty interval
YLDs: Years lost to disability

## References

[1] Cieza A, Causey K, Kamenov K, Hanson SW, Chatterji S, Vos T (2021). Global estimates of the need for rehabilitation based on the Global Burden of Disease study 2019: a systematic analysis for the Global Burden of Disease Study 2019. Lancet.

[2] Australian Burden of Disease Study: impact and causes of illness and death in Australia 2015—summary report. Australian Burden of Disease Study series no. 18. Cat. no. BOD 21. In: AIHW, editor. Canberra: AIHW; 2019.

[3] Walker BF, Muller R, Grant WD (2004). Low back pain in Australian adults: prevalence and associated disability. J Manipulative Physiol Ther.

[4] Deloitte Access Economics. The cost of pain in Australia. Painaustralia; 2019.

[5] Britt H, Miller GC, Henderson J (2015). General practice series no. 38.

[6] Schofield DJ, Shrestha RN, Cunich M, Tanton R, Kelly S, Passey ME (2015). Lost productive life years caused by chronic conditions in Australians aged 45–64 years, 2010–2030. Med J Aust.

[7] AIHW. Back problems. Canberra: AIHW; 2020.

[8] Vos T, Lim SS, Abbafati C, Abbas KM, Abbasi M, Abbasifard M (2020). Global burden of 369 diseases and injuries in 204 countries and territories, 1990–2019: a systematic analysis for the Global Burden of Disease Study 2019. Lancet.

[9] Safiri S, Kolahi AA, Cross M, Carson-Chahhoud K, Almasi-Hashiani A, Kaufman J (2021). Global, regional, and national burden of other musculoskeletal disorders 1990–2017: results from the Global Burden of Disease Study 2017. Rheumatology (Oxford).

[10] Safiri S, Kolahi AA, Hoy D, Buchbinder R, Mansournia MA, Bettampadi D (2020). Global, regional, and national burden of neck pain in the general population, 1990–2017: systematic analysis of the Global Burden of Disease Study 2017. BMJ.

[11] Safiri S, Kolahi AA, Hoy D, Smith E, Bettampadi D, Mansournia MA (2019). Global, regional and national burden of rheumatoid arthritis 1990–2017: a systematic analysis of the Global Burden of Disease study 2017. Ann Rheum Dis.

[12] Safiri S, Kolahi AA, Smith E, Hill C, Bettampadi D, Mansournia MA (2020). Global, regional and national burden of osteoarthritis 1990–2017: a systematic analysis of the Global Burden of Disease Study 2017. Ann Rheum Dis.

[13] Wu A, March L, Zheng X, Huang J, Wang X, Zhao J (2020). Global low back pain prevalence and years lived with disability from 1990 to 2017: estimates from the Global Burden of Disease Study 2017. Ann Transl Med.

[14] Wu A, Dong W, Liu S, Cheung JPY, Kwan KYH, Zeng X (2019). The prevalence and years lived with disability caused by low back pain in China, 1990 to 2016: findings from the global burden of disease study 2016. Pain.

[15] Shahrezaee M, Keshtkari S, Moradi-Lakeh M, Abbasifard M, Alipour V, Amini S (2020). Burden of musculoskeletal disorders in Iran during 1990–2017: estimates from the Global Burden of Disease Study 2017. Arch Osteoporos.

[16] Clark P, Denova-Gutiérrez E, Razo C, Rios-Blancas MJ, Lozano R (2018). The burden of musculoskeletal disorders in Mexico at national and state level, 1990–2016: estimates from the global burden of disease study 2016. Osteoporos Int.

[17] Lundkvist A, El-Khatib Z, Kalra N, Pantoja T, Leach-Kemon K, Gapp C (2021). Policy-makers' views on translating burden of disease estimates in health policies: bridging the gap through data visualization. Arch Public Health.

[18] Mathers CD (2020). History of global burden of disease assessment at the World Health Organization. Arch Public Health.

[19] Stevens GA, Alkema L, Black RE, Boerma JT, Collins GS, Ezzati M (2016). Guidelines for accurate and transparent health estimates reporting: the GATHER statement. Lancet.

[20] Hoy D, March L, Brooks P, Blyth F, Woolf A, Bain C (2014). The global burden of low back pain: estimates from the Global Burden of Disease 2010 study. Ann Rheum Dis.

[21] Hoy D, March L, Woolf A, Blyth F, Brooks P, Smith E (2014). The global burden of neck pain: estimates from the global burden of disease 2010 study. Ann Rheum Dis.

[22] Hoy DG, Smith E, Cross M, Sanchez-Riera L, Buchbinder R, Blyth FM (2014). The global burden of musculoskeletal conditions for 2010: an overview of methods. Ann Rheum Dis.

[23] GBD 2017 Causes of Death Collaborators. Global, regional, and national age-sex-specific mortality for 282 causes of death in 195 countries and territories, 1980–2017: a systematic analysis for the Global Burden of Disease Study 2017. Lancet. 2018;392(10159):1736–88. 10.1016/S0140-6736(18)32203-7 PMC6227606 30496103

[24] Global, regional, and national age-sex-specific mortality and life expectancy, 1950–2017: a systematic analysis for the Global Burden of Disease Study 2017. Lancet. 2018;392(10159):1684–735. 10.1016/S0140-6736(18)31891-9 PMC6227504 30496102

[25] Collaborators. GRF. Global burden of 87 risk factors in 204 countries and territories, 1990–2019: a systematic analysis for the Global Burden of Disease Study 2019. . The Lancet; 2020. 10.1016/S0140-6736(20)30752-2 PMC7566194 33069327

[26] Briggs AM, Huckel Schneider C, Slater H, et al. Health systems strengthening to arrest the global disability burden: empirical development of prioritised components for a global strategy for improving musculoskeletal health BMJ Glob Health; 2021. 10.1136/bmjgh-2021-006045 PMC8215245 37904582

[27] AIHW. Australian Burden of Disease Study: Impact and causes of illness and death in Australia 2018. Canberra: Australian Government; 2021.

[28] Zhao C, Choi C, Laws P, Gourley M, Dobson A, Driscoll T, et al. Value of a national burden-of-disease study: a comparison of estimates between the Australian Burden of Disease Study 2015 and the Global Burden of Disease Study 2017. Int J Epidemiol. 2021. 10.1093/ije/dyab093 34058000

[29] O'Sullivan PB, Beales DJ, Smith AJ, Straker LM (2012). Low back pain in 17 year olds has substantial impact and represents an important public health disorder: a cross-sectional study. BMC Public Health.

[30] Macfarlane GJ, Beasley M, Jones EA, Prescott GJ, Docking R, Keeley P (2012). The prevalence and management of low back pain across adulthood: results from a population-based cross-sectional study (the MUSICIAN study). Pain.

[31] Lentz TA, Harman JS, Marlow NM, Beneciuk JM, Fillingim RB, George SZ (2019). Factors associated with persistently high-cost health care utilization for musculoskeletal pain. PLoS ONE.

[32] Palmer KT, Syddall H, Cooper C, Coggon D (2003). Smoking and musculoskeletal disorders: findings from a British national survey. Ann Rheum Dis.

[33] Eriksen WB, Brage S, Bruusgaard D (1997). Does smoking aggravate musculoskeletal pain?. Scand J Rheumatol.

[34] De Carvalho DE, de Luca K, Funabashi M, Breen A, Wong AYL, Johansson MS (2020). Association of exposures to seated postures with immediate increases in back pain: a systematic review of studies with objectively measured sitting time. J Manip Physiol Ther.

[35] Coenen P, Gouttebarge V, van der Burght AS, van Dieën JH, Frings-Dresen MH, van der Beek AJ (2014). The effect of lifting during work on low back pain: a health impact assessment based on a meta-analysis. Occup Environ Med.

[36] Wynne-Jones G, Cowen J, Jordan JL, Uthman O, Main CJ, Glozier N (2014). Absence from work and return to work in people with back pain: a systematic review and meta-analysis. Occup Environ Med.

[37] Nieminen LK, Pyysalo LM, Kankaanpää MJ (2021). Prognostic factors for pain chronicity in low back pain: a systematic review. Pain Rep.

[38] Felício DC, Filho JE, de Oliveira TMD, Pereira DS, Rocha VTM, Barbosa JMM, et al. Risk factors for non-specific low back pain in older people: a systematic review with meta-analysis. Arch Orthop Trauma Surg. 2021. 10.1007/s00402-021-03959-0 34021388

[39] Speerin R, Needs C, Chua J, Woodhouse LJ, Nordin M, McGlasson R (2020). Implementing models of care for musculoskeletal conditions in health systems to support value-based care. Best Pract Res Clin Rheumatol.

[40] Lin I, Wiles L, Waller R, Goucke R, Nagree Y, Gibberd M (2020). What does best practice care for musculoskeletal pain look like? Eleven consistent recommendations from high-quality clinical practice guidelines: systematic review. Br J Sports Med.

[41] Australian Government Department of Health. National strategic action plan for pain management. Canberra, ACT, Australia: Commonwealth of Australia; 2019.

[42] Tamrakar M, Kharel P, Traeger A, Maher C, O'Keeffe M, Ferreira G (2021). Completeness and quality of low back pain prevalence data in the Global Burden of Disease Study 2017. BMJ Glob Health.

[43] Hoy DG, Raikoti T, Smith E, Tuzakana A, Gill T, Matikarai K (2018). Use of The Global Alliance for Musculoskeletal Health survey module for estimating the population prevalence of musculoskeletal pain: findings from the Solomon Islands. BMC Musculoskelet Disord.

