# Supplementary material for: Disability burden due to musculoskeletal conditions and low back pain in Australia: findings from GBD 2019
Source: Chiropr Man Therap. 2022 May 3;30:22. doi: 10.1186/s12998-022-00434-4 (PMC9063272; doi:10.1186/s12998-022-00434-4)
Supplement: Supplementary file 1 — Additional file 1. GBD lay descriptions and disability weights for low back pain in 2010 and 2019. [file 12998_2022_434_MOESM1_ESM.docx]

Additional file 1: GBD lay descriptions and disability weights for low back pain in 2010 and 2019.(8)

| **Severity level** | **Lay description** | **Disability weights** | **Disability weights** |
| --- | --- | --- | --- |
|  |  | **GBD 2010** | **GBD 2019** |
| Low back pain, mild | This person has mild back pain, which causes some difficulty dressing, standing and lifting things. | Not included | 0.020 (0.011-0.035) |
| Low back pain, moderate | This person has moderate back pain, which causes difficulty dressing, sitting, standing, walking and lifting things. | Not included | 0.054 (0.035-0.079) |
| Low back pain, severe without leg pain  *Severe acute low back pain without leg pain* | This person has severe *low* back pain, which causes difficulty dressing, sitting, standing, walking and lifting things. The person sleeps poorly and feels worried. | 0.269 (0.184-0.373) | 0.272 (0.182-0.373) |
| Low back pain, severe with leg pain  *Severe acute low back pain with leg pain* | This person has severe *low* back and leg pain, which causes difficulty dressing, sitting, standing, walking and lifting things. The person sleeps poorly and feels worried. | 0.322 (0.219-0.447) | 0.325 (0.219-0.446) |
| Low back pain, most severe without leg pain  *Severe chronic low back pain without leg pain* | This person has constant *low* back pain, which causes difficulty dressing, sitting, standing, walking and lifting things. The person sleeps poorly, is worried, and has lost some enjoyment in life. | 0.366 (0.248-0.499) | 0.372 (0.250-0.506) |
| Low back pain, most severe with leg pain  *Severe chronic low back pain with leg pain* | This person has constant *low* back and leg pain, which causes difficulty dressing, sitting, standing, walking and lifting things. The person sleeps poorly, is worried, and has lost some enjoyment in life. | 0.374 (0.252-0.506) | 0.384 (0.256-0.518) |
| 95% uncertainty intervals are in parentheses | | | |
| * Differences in lay descriptions between GBD 2010 and GBD 2019 are shown, where the italicised text is the lay description from GBD 2010. | | | |
