# Supplementary material for: Disability burden due to musculoskeletal conditions and low back pain in Australia: findings from GBD 2019
Source: Chiropr Man Therap. 2022 May 3;30:22. doi: 10.1186/s12998-022-00434-4 (PMC9063272; doi:10.1186/s12998-022-00434-4)
Supplement: Supplementary file 2 — Additional file 2. Proportions within severity level, split by with and without leg pain, for low back pain in GBD 2010 and GBD 2019. [file 12998_2022_434_MOESM2_ESM.docx]

Additional file 2: Proportions within severity level, split by with and without leg pain, for low back pain in GBD 2010 and GBD 2019.(8)

| **Severity level** | **Proportions without leg pain** | | **Proportions with leg pain** | |
| --- | --- | --- | --- | --- |
|  | GBD 2010 | GBD 2019 | GBD 2010 | GBD 2019 |
| Low back pain, mild  *Mild acute* | 0.49 (0.42-0.57) | 0.41 (0.31-0.53) | 0.36 (0.28-0.44) | 0.27 (0.19-0.37) |
| Low back pain, moderate  *Mild chronic* | 0.23 (0.17-0.29) | 0.35 (0.25-0.44) | 0.26 (0.20-0.33) | 0.36 (0.28-0.43) |
| Low back pain, severe  *Severe acute* | 0.11 (0.08-0.13) | 0.10 (0.08-0.12) | 0.12 (0.09-0.15) | 0.14 (0.10-0.16) |
| Low back pain, most severe  *Severe chronic* | 0.17 (0.12-0.23) | 0.14 (0.09-0.20) | 0.26 (0.18-0.34) | 0.23 (0.15-0.32) |
| 95% uncertainty intervals are in parentheses | | | | |
| * Differences in lay proportions between GBD 2010 and GBD 2019 are shown, where the italicised text is the lay description from GBD 2010. | | | | |
